# Healthcare utilization and costs in patients beginning pharmacotherapy for generalized anxiety disorder: a retrospective cohort study

**DOI:** 10.1186/1471-244X-11-193

**Published:** 2011-12-12

**Authors:** Ariel Berger, John Edelsberg, Vamsi Bollu, Jose Ma J Alvir, Ashish Dugar, Ashish V Joshi, Gerry Oster

**Affiliations:** 1 Policy Analysis Inc. (PAI), Four Davis Court, Brookline, MA, 02445, USA; 2 Novartis Pharmaceuticals Corporation, Bldg 432/554-2D, One Health Plaza, East Hanover, NJ, 07045 USA; 3 Pfizer Inc., 235 East 42nd Street, New York, NY, 10017, USA

## Abstract

**Background:**

Patterns of healthcare utilization and costs in patients beginning pharmacotherapy for generalized anxiety disorder (GAD) have not been well characterized.

**Methods:**

Using a large US health insurance database, we identified all patients with evidence of GAD (ICD-9-CM diagnosis code 300.02) who initiated pharmacotherapy with medications commonly used to treat GAD (eg, selective serotonin reuptake inhibitors [SSRIs], venlafaxine, benzodiazepines) between 1/1/2003 and 12/31/2007. We examined healthcare utilization and costs over the 12-month periods preceding and following date of initial receipt of such therapy ("pretreatment" and "follow-up", respectively). Patients with incomplete data were excluded.

**Results:**

A total of 10,275 patients met all study inclusion criteria. Forty-eight percent of patients received SSRIs; 34%, benzodiazepines; and 6%, venlafaxine. SSRIs and venlafaxine were about three times more likely to be used on a long-term basis (> 90 days) than benzodiazepines (p < 0.01). In general, levels of healthcare utilization were higher during follow-up than pretreatment. Mean (SD) total healthcare costs increased from $4812 ($10,006) during pretreatment to $7182 ($22,041) during follow-up (p < 0.01); costs of GAD-related pharmacotherapy during follow-up were $420 ($485).

**Conclusions:**

More than one-half of patients initiating pharmacotherapy for GAD receive either SSRIs or venlafaxine. Levels of healthcare utilization and costs are greater in the year following initiation of therapy than in the immediately preceding one.

## Background

Generalized anxiety disorder (GAD) is a chronic condition characterized by persistent worry or anxiety [1]; it is often difficult to diagnose because of the wide variety of clinical presentations and the common occurrence of comorbid somatic diseases and/or mental disorders. Lifetime prevalence of GAD has been estimated to range from 4% to 6% [2]; annual prevalence has been reported to be about 2% [3,4]. GAD is two to three times more common in women than men [3]. GAD is the most common anxiety disorder among patients presenting to primary care physicians [5,6], and it is overrepresented in primary care settings, with point prevalence rates at least 2-3 times higher than those reported in the community [6,7]. GAD typically follows a relapsing/remitting pattern; approximately one-third of patients who achieve remission experience a full relapse within three years [8].

Recent clinical guidelines recommend first-line treatment with antidepressants--specifically, escitalopram, paroxetine, or sertraline (all selective serotonin re-uptake inhibitors [SSRI]), or venlafaxine (a serotonin-norepinephrine re-uptake inhibitor [SNRI])--on the basis of their efficacy, safety, and tolerability [9,10]. While benzodiazepines were the mainstay of GAD treatment for many years because of their favorable tolerability and the rapid symptomatic relief that they typically provide, there is general agreement today that--excepting patients who are refractory to other available therapies--they should not be used for more than a few weeks, due to risks of dependency and sedation, increased risk of industrial and motor vehicle accidents, and neonatal and infant mortality when used in late pregnancy or during breast feeding [11,12]. A substantial proportion of patients receiving benzodiazepines also develop rebound anxiety, an intensification of previous symptoms, or withdrawal when treatment is discontinued [13-15]. On the other hand, antidepressants also are effective in treating comorbid depression that is common in patients with GAD, and there is evidence that they may be more effective than benzodiazepine anxiolytics on the psychic symptoms of anxiety.

Utilization of pharmacotherapy for GAD in real-world clinical practice has not been extensively studied. A recent study based on health insurance claims reported that total healthcare expenditures increased by $1340 between the 12-month periods before and after a diagnosis of GAD was first rendered [16]. The few available other studies have been limited by narrow geographical focus, failure to distinguish patients with GAD from those with other anxiety disorders, and--in some cases--relatively small numbers of patients [17-19]. In this study, we investigate patterns of initial pharmacotherapy for GAD in a large, geographically diverse population, and changes in healthcare utilization and costs in the periods immediately before and after initiation of such therapy.

## Methods

### Data Source

Data were obtained from the PharMetrics Patient-Centric Database. The database is comprised of facility, professional-service, and retail (i.e., outpatient) pharmacy claims from over 85 health plans. The plans provide healthcare coverage to approximately 14 million persons annually throughout the US (Midwest, 35%; Northeast, 21%; South, 31%; West, 13%). All patient identifiers in the database have been fully encrypted, and the database is fully compliant with the Health Insurance Portability and Accountability Act of 1996 (HIPAA).

Information available for each facility and professional-service claim includes date and place of service, diagnoses (in ICD-9-CM format), procedures (in ICD-9-CM [selected plans only] and HCPCS formats), provider specialty, and charged and paid amounts. Data available for each retail pharmacy claim include the drug dispensed (in NDC format), the dispensing date, and the quantity dispensed and number of days of therapy supplied (selected plans only). All claims include a charged amount; the database also provides paid (i.e., reimbursed, including patient deductible, copayment, and/or coinsurance) amounts.

Selected demographic and eligibility information is also available, including age, gender, geographic region, coverage type, and the dates of insurance coverage. All patient-level data can be arrayed in chronologic order to provide a detailed, longitudinal profile of all medical and pharmacy services used by each plan member. The database for this study encompassed the period, January 1, 2003 through December 31, 2007 ("study period").

### Study Sample

The source population for our study consisted of all persons with two or more outpatient claims on different days (during the study period) with a diagnosis of GAD (ICD-9-CM diagnosis code 300.02). Among these patients, we identified those initiating pharmacotherapy with any of a number of medications that are often used to treat GAD ("GAD-related medications"), as follows: (1) SSRIs (escitalopram, paroxetine, sertraline); (2) venlafaxine (an SNRI); (3) benzodiazepines (alprazolam, chlordiazepoxide, clonazepam, clorazepate, diazepam, lorazepam, oxazepam); and (4) other agents (imipramine, buspirone, hydroxyzine, trifluoperazine) [9,19]. While fluoxetine, an SSRI, is sometimes used to treat GAD, we did not include it because it is not indicated for the treatment of GAD, and because there is little or no evidence from clinical trials supporting its use in this indication. Date of initial receipt of a GAD-related medication was designated the "index date".

Patients without at least one claim with a diagnosis code of GAD in the 90-day period immediately preceding (and including) their index date were excluded from the study sample, as were: (1) patients with less than 12 months of complete data prior to their index date ("pretreatment"); (2) patients with less than 12 months of complete data subsequent to their index date ("follow-up"); (3) patients with evidence of receipt during the pretreatment period of any medications commonly used to treat GAD (as noted above) or any other medication from any such class; (4) Medicaid beneficiaries; or (5) patients aged ≥ 65 years who were enrolled in a Medicare supplemental or fee-for-service plan (their claims histories may be incomplete). For all remaining patients, we compiled all pharmacy, professional service, and facility claims during both the pretreatment and follow-up periods.

Patients were then stratified into treatment groups based on the agent received on the index date ("initial therapy"). Patients with evidence of receipt of more than one GAD-related agent on their index date (e.g., SSRI and a benzodiazepine) were assigned to a "combination therapy" group.

### Measures and Analyses

Baseline demographic and clinical characteristics of study subjects, including prevalence of selected comorbidities (Additional File 1), were characterized on the basis of information during the 12-month pretreatment period.

Use of GAD-related medication was examined in terms of the numbers of patients receiving various medications (by drug class and agent), as well as the numbers of pharmacy claims for--and associated therapy-days with--each medication.

Duration of receipt of initial therapy was defined based on time between the index date and the date of final receipt of such therapy, where the latter was designated based on the first pharmacy claim that was followed by a ≥ 28-day "gap" between the final therapy-day associated with that claim and the date of the next claim (if any) for such therapy. Thus, for example, if a patient's date of initial receipt of an SSRI was January 1 and 28 days of such therapy were dispensed, the last day of treatment was assumed to have been January 28 unless there was another pharmacy claim for the same agent on or before February 26 (i.e., 28 days subsequent to January 28). If the patient had another claim for the same SSRI within this timeframe, a similar rule was applied to the next pharmacy claim, and so on.

We examined healthcare utilization during the pretreatment and follow-up periods in terms of the numbers of physician office visits, other outpatient office visits, emergency department (ED) visits, and hospitalizations. Length of stay (LOS) also was examined for patients admitted to hospital. Total healthcare costs were tallied in terms of: (1) GAD-related medications (initial therapy as well as all other such agents received during follow-up); (2) all other pharmacotherapy; (3) physician office visits; (4) other outpatient visits; (5) ED visits; (6) inpatient care; and (7) all other care. Reimbursed amounts (including any patient liability, such as co-pays and co-insurance) were used in all analyses of health-care costs. Healthcare utilization and costs were characterized on an overall basis and by month during the pretreatment and follow-up periods, respectively.

The statistical significance of differences between pretreatment and follow-up was assessed using paired t-tests for continuous variables with approximately normal distributions, and Wilcoxon signed-rank tests otherwise. McNemar and Bowker's tests were used to assess the statistical significance of differences in categorical variables, as appropriate. All tests of statistical significance were two-tailed with an alpha level of 0.05. All analyses were conducted using SAS^® ^Proprietary Software, Release 9.1 (SAS Institute Inc., Cary, NC).

## Results

We identified 10,275 patients with GAD who initiated treatment with one of the agents of interest and who also met all other study entry criteria (Table 1). Mean age was 37.7 years, and 60% of study subjects were women (Table 2). Thirty-eight percent of study subjects received diagnoses of depressive disorders during pretreatment, and 18% had diagnoses of other anxiety disorders.

**Table 1:** Selection of study subjects

	Patients
Number of patients with ≥ 2 outpatient claims for GAD and	285,820

≥ 1 claims for pharmacotherapy used to treat GAD and	167,538

≥ 1 claims with GAD diagnosis on index date or during 90-day period immediately prior and	55,791

≥ 12 months enrollment prior to index date* and	22,678

≥ 12 months enrollment following index date* and	14,407

No evidence of receipt of any benzodiazepine, SSRI or SNRI in the year prior to index date and**	11,202

No Medicaid insurance and	11,111

Aged < 65 years or	10,398

Aged ≥ 65 years and not enrolled in Medicare supplemental or capitated plans	119

Total of above and	10,517

No missing information on therapy-days on prescription for index drug	10,275

*First-noted claim for medication of interest during study period

**Including agents that are and are not recommended for use in GAD

GAD: generalized anxiety disorder; SSRI: Selective serotonin reuptake inhibitor; SNRI: Serotonin-norepinephrine reuptake inhibitor

**Table 2:** Demographic and clinical characteristics of study subjects (N = 10,275)*

Characteristic		
Age, years		

< 18	1,114	(10.8)

18-44	5,506	(53.6)

45-54	2,176	(21.2)

55-64	1,363	(13.3)

≥ 65	116	(1.1)

Mean (SD)	37.7	(14.9)

Female	6,155	(59.9)

Comorbidities		

Mental disorders		

Other anxiety disorders	1,813	(17.6)

Depressive disorders	3,885	(37.8)

Bipolar disorder	63	(0.6)

Tension headache	114	(1.1)

Personality disorders	101	(1.0)

Alcohol abuse/alcoholism	19	(0.2)

Drug abuse	132	(1.3)

Suicide attempts	97	(0.9)

Sleep disorders	1,134	(11.0)

Neoplasms	252	(2.5)

Diabetes	396	(3.9)

Migraine	414	(4.0)

Ischemic heart disease	280	(2.7)

Cerebrovascular disease	138	(1.3)

Asthma	664	(6.5)

Painful neuropathic disorders	964	(9.4)

Symptoms, signs, and ill- defined conditions		

Fatigue	1,690	(16.4)

Headache	974	(9.5)

Chest pain	1,431	(13.9)

Abdominal pain	1,552	(15.1)

Anxiety- related symptoms	1,627	(15.8)

Any symptoms, signs, and ill- defined conditions	4,692	(45.7)

Pretreatment healthcare costs, $		

Mean (SD)	4,812	(10,006)

Median (IQR)	2,118	(896,4,975)

Region		

Northeast	3,165	(30.8)

South	1,621	(15.8)

West	1,858	(18.1)

Midwest	3,631	(35.3)

Payer type		

HMO	2,971	(28.9)

PPO	4,733	(46.1)

Indemnity	554	(5.4)

Other	283	(2.8)

*Unless otherwise indicated, all values are number (%)

GAD: Generalized anxiety disorder; SAD: Social anxiety disorder; HMO: Health maintenance organization; IQR: Interquartile range; PPO: Preferred provider organization; SD: Standard deviation

SSRIs were the most widely used agents (47.6% of study subjects received an SSRI on their index date), followed by the benzodiazepines (34.0%), and venlafaxine (6.3%); 6.6% of patients received combination therapy on the index date. Among SSRIs, the most commonly used agents were escitalopram (20.1% of study subjects) and sertraline (17.5%). Among benzodiazepines, the most commonly used agents were alprazolam (17.2%) and lorazepam (7.4%).

Patterns of pharmacotherapy differed substantially by type of agent received. Patients who initiated therapy with an SSRI received an average (SD) of 5.9 (4.0) prescriptions; corresponding values for venlafaxine, benzodiazepines, and other GAD-related medications were 5.8 (4.3), 3.5 (3.6), and 2.6 (2.7), respectively. Patients who received combination therapy on the index date had an average of 9.9 (6.6) prescriptions over 12 months. Mean (SD) duration of therapy was 24.6 (32.5) days among patients initiating therapy with benzodiazepines; corresponding figures for venlafaxine, SSRIs, and other GAD-related medications were 75.5 (64.8) days, 70.4 (59.8) days, and 32.0 (34.8) days, respectively. Among those receiving a combination regimen on their index date, mean duration was 78.0 (60.2) days.

**Figure 1:**
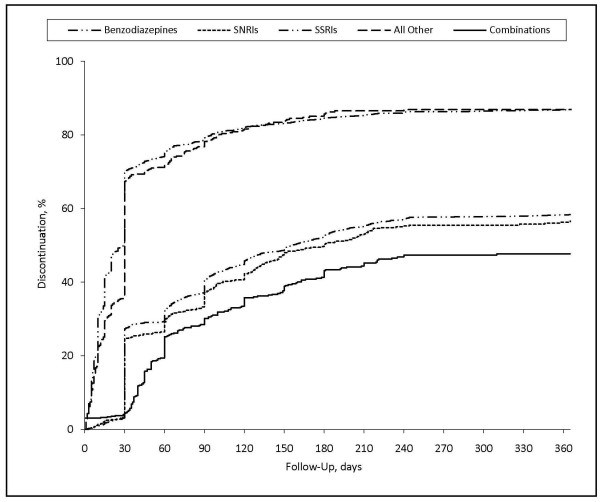
**Discontinuation of initial therapy for GAD**. GAD: Generalized anxiety disorder; SNRI: Serotonin-norepinephrine reuptake inhibitor; SSRI: Selective serotonin reuptake inhibitor.

SSRIs and venlafaxine were more likely than benzodiazepines to be used on a long-term basis (> 90 days) (Figure 1). At one year, 41.7% and 43.4% of SSRI and venlafaxine patients were still receiving treatment; the corresponding value for benzodiazepine patients was 13.2%.

**Figure 2:**
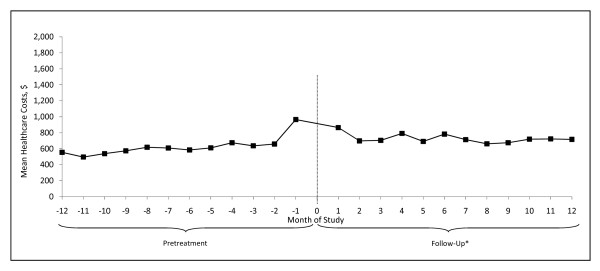
**Mean total healthcare costs during pretreatment and follow-up**. *Includes index date.

In general, levels of healthcare utilization were higher during follow-up than pretreatment. Of particular note, there was an increase in the number of physician office visits, from a mean (SD) of 12.6 (13.2) during pretreatment to 16.3 (15.5) during follow-up (Table 3). Mean total healthcare costs increased by $2370 between pretreatment and follow-up (p < 0.01) (Table 4). Costs of GAD-related pharmacotherapy were $420 ($485) during follow-up. Costs were highest around the time of therapy initiation (Figure 2).

**Table 3:** Use of healthcare services during pretreatment and follow-up

	Pretreatment	Follow-Up	*P*-Value
Office visits			

Psychiatrists			

Mean (SD)	1.0 (3.3)	2.1 (5.1)	< 0.01

Median (IQR)	0 (0, 0)	0 (0, 2)	

Internists			

Mean (SD)	0.9 (2.3)	1.1 (2.9)	0.01

Median (IQR)	0 (0, 1)	0 (0, 1)	

General practitioners			

Mean (SD)	1.9 (3.3)	2.4 (3.7)	< 0.01

Median (IQR)	1 (0, 3)	1 (0, 4)	

Other			

Mean (SD)	9.1 (12.1)	11.1 (14.2)	< 0.01

Median (IQR)	5 (2, 12)	6 (2, 15)	

Total			

Mean (SD)	12.6 (13.2)	16.3 (15.5)	< 0.01

Median (IQR)	8 (4, 16)	11 (6, 21)	

Other outpatient office visits			

Mean (SD)	1.3 (2.6)	1.5 (3.5)	0.01

Median (IQR)	0 (0, 2)	0 (0, 2)	

Total outpatient office visits			

Mean (SD)	13.5 (13.6)	17.3 (16.0)	< 0.01

Median (IQR)	9 (5, 17)	12 (6, 23)	

ED visits			

Mean (SD)	0.3 (1.1)	0.3 (1.1)	0.28

Median (IQR)	0 (0, 0)	0 (0, 0)	

Hospitalizations			

Mean (SD)	0.3 (2.0)	0.4 (2.4)	0.04

Median (IQR)	0 (0, 0)	0 (0, 0)	

GAD: Generalized anxiety disorder; SD: Standard deviation; ED: Emergency department; IQR: Interquartile range

**Table 4:** Mean healthcare costs during pretreatment and follow-up

	Pretreatment	Follow-Up	*P*-Value
			

Pharmacotherapy			

GAD related			

SSRIs	0 (0)	302 (385)	

SNRIs	0 (0)	82 (331)	

Benzodiazepines	0 (0)	28 (119)	

Other	0 (0)	8 (55)	

Total of above	0 (0)	420 (485)	

All other	639 (1,416)	1,128 (2,463)	< 0.01

Total of above	639 (1,416)	1,548 (2,552)	< 0.01

Outpatient care			

Physician's office visits			

Psychiatrists	117 (434)	220 (660)	< 0.01

Internists	90 (282)	117 (508)	0.03

General practitioners	170 (387)	217 (432)	< 0.01

All other	1,135 (1,916)	1,444 (2,559)	< 0.01

Total of above	1,511 (2,110)	1,997 (2,863)	< 0.01

Other outpatient office vists	747 (2,527)	1,101 (5,021)	< 0.01

Total of above	2,258 (3,628)	3,098 (6,357)	< 0.01

ED	236 (900)	279 (1,152)	0.20

Inpatient care	1,173 (7,433)	1,573 (17,214)	0.05

All other	506 (1,913)	684 (3,514)	< 0.01

Total	4,812 (10,006)	7,182 (22,041)	< 0.01

*All values are mean $ (SD)

GAD: Generalized anxiety disorder; SSRI: Selective serotonin reuptake inhibitor; SNRI: Serotonin-norepinephrine reuptake inhibitor; ED: Emergency department; SD: Standard deviation

## Discussion

About one-half of all patients beginning a new course of pharmacotherapy for GAD receive either an SSRI or venlafaxine. The typical duration of therapy with these agents was only about three months; almost one-half of patients who began treatment with an SSRI or venlafaxine, however, had evidence of continuing receipt of medication at one year. Benzodiazepines, which about one-third of study subjects received as initial therapy, were administered--not unexpectedly--for a shorter period time, and mean duration of therapy was about one month.

About one in five patients beginning treatment with a benzodiazepine had evidence of continuing receipt beyond 90 days. Lacking information on disease severity, we could not assess the appropriateness of such long-term use. To the extent these patients had symptoms refractory to other medications, long-term treatment with benzodiazepines may have been appropriate, notwithstanding well-known risks associated with such therapy (e.g., dependence, cognitive impairment, increased risk of falls, risk of rebound anxiety) when treatment is discontinued [11-15]).

Mean total healthcare costs were $2370 higher during the one-year period of follow-up than in the one-year period preceding treatment. While we do not know the exact reason(s) for this difference, we suspect that it may be related to GAD-related care (GAD-related pharmacotherapy comprised 18% of this increase), exacerbation of pre-existing comorbidities or decreased ability to cope with these conditions, and/or new somatic manifestations of GAD (e.g., chest pain, gastrointestinal disorders). Further research is needed to better understand this phenomenon.

The increase in costs (follow-up vs pretreatment) that we observed is substantially greater than that reported by Francois and colleagues ($1340). We suspect that the principal reason for this difference is that our study only included patients initiating pharmacotherapy for GAD, while only 44% of the patients in the Francois study received an antidepressant or anxiolytic anytime during follow-up [16]. Presumably, patients initiating pharmacotherapy for GAD will have more severe illness--and incur greater costs--than patients newly diagnosed with GAD who do not receive drug therapy.

An important limitation of our study was the omission of fluoxetine from the group of SSRIs. We excluded fluoxetine because it is not indicated for the treatment of GAD, and has not been studied extensively in this indication. Nonetheless, there is evidence that it may be prescribed for this disorder [20-23]. In one recent study of 305 patients with GAD and 232 with social phobia, it was reported to be the most commonly prescribed SSRI [18] (benzodiazepines were the most commonly used medications in both indications). Our exclusion of fluoxetine undoubtedly resulted in our underestimating the number of GAD patients beginning therapy with an SSRI. We note, however, that although fluoxetine was not on our list of designated GAD-related medications, patients who received it during the one-year pretreatment period were excluded from our study sample (as were those who received any other SSRI, any SNRI, or any benzodiazepine during pretreatment). Thus, patients could not have been included in our study population if they were adding a second agent (e.g., a benzodiazepine) to fluoxetine or switching from fluoxetine to another agent.

There are several other important limitations of our study. First, as with all claims database studies, there may be errors of omission and commission in coding. Accordingly, some patients with GAD may not have been included in our study sample due to absence of appropriate diagnoses on healthcare claims, while others who received a diagnosis of GAD incorrectly and therefore should have been excluded were not. As patient medical records were not available to us, the degree to which patients were actually misclassified is unknown. Also, the database does not contain important clinical information on disease severity. Without such information, it is difficult to assess the appropriateness of the therapeutic patterns that we observed.

## Conclusions

In conclusion, about one-half of all patients beginning a new course of pharmacotherapy for GAD receive either an SSRI or venlafaxine, and roughly one-third receive a benzodiazepine. Levels of healthcare utilization and costs are greater in the year following initiation of therapy than in the immediately preceding one.

## Declaration of competing interests

Mr. Berger, Dr. Edelsberg, and Dr. Oster are employed by Policy Analysis Inc., an independent contract research organization with previous and ongoing engagements with Pfizer Inc. as well as other pharmaceutical manufacturers. Drs. Alvir, Dugar, and Joshi are employed by Pfizer Inc.; at the time the work was undertaken, Dr. Bollu also was employed by Pfizer Inc.

## Authors' contributions

All authors reviewed and contributed to the study research plan, interpretation of the data, and the study manuscript; data management, processing, and analyses were conducted by AB, JE, and GO. All authors read and approved the final manuscript.

## Pre-publication history

The pre-publication history for this paper can be accessed here:

http://www.biomedcentral.com/1471-244X/11/193/prepub

## Supplementary Material

Additional file 1 **Definitions of comorbidities of interest**. Click here for file
