# Supplementary material for: Healthcare utilization and costs in patients beginning pharmacotherapy for generalized anxiety disorder: a retrospective cohort study
Source: BMC Psychiatry. 2011 Dec 12;11:193. doi: 10.1186/1471-244X-11-193 (PMC3266199; doi:10.1186/1471-244X-11-193)
Supplement: Additional file 1 — Definitions of comorbidities of interest. [file 1471-244X-11-193-S1.DOC]

| **Comorbidity** | | |  | **ICD-9-CM  Diagnosis Codes** |  | **Medications** |
| --- | --- | --- | --- | --- | --- | --- |
| Mental disorders | | |  |  |  |  |
|  | Other anxiety disorders | |  | 300.01, 300.3, 309.81, 300.2X (except where specified elsewhere), 293.84, 309.21, 300.23, 300.0, 300.00, 300.09, 300.1X |  |  |
|  | Depressive disorders | |  | 300.4, 309.0, 309.1, 296.2, 296.3, 311.XX, 290.21, 292.84, 296.20-296.XX, 298.0 |  |  |
|  | Bipolar disorder | |  | 296.0X, 296.4X, 296.5X, 296.6X, 296.7X, 296.8X (except for bipolar depression cohort) |  |  |
|  | Tension headache | |  | 307.81 |  |  |
|  | Personality disorders | |  | 301.XX |  |  |
|  | Alcohol abuse/alcoholism | |  | 291.0, 291.1, 291.2, 291.3, 291.5, 291.89, 291.9, 303.0, 303.9, 305.0, 571.2, 648.4, V11.3 |  |  |
|  | Drug abuse | |  | 304.0-304.9, 305.2-305.9 |  |  |
|  | Suicide attempts | |  | 300.9, E950-E959 |  |  |
| All other | | |  |  |  |  |
|  | Sleep disorders | |  | 780.51, 780.52, 307.41, 307.42, 307.49, 780.53, 780.57, 786.03, 347.0X, 347.1X, V69.4, 780.5, 780.50, 780.54, 780.55, 780.56, 780.58, 780.59 |  |  |
|  | Neoplasms (except squamous or basal cell skin carcinoma) | |  | 140.XX-172.XX, 174.XX-208.XX |  |  |
|  | Diabetes | |  | 250.XX |  | Alpha-glucosidase inhibitors, insulin, metformin, nonsulfonylurea insulin secretagogues, sulfonylurea or thiazolidinedione |
|  | Migraine | |  | 346.XX |  |  |
|  | Ischemic heart disease | |  | 410-414.XX |  |  |
|  | Cerebrovascular disease | |  | 430-438.XX |  |  |
|  | Asthma | |  | 493.XX |  |  |
|  | Painful neuropathic disorders | |  | 250.6X, 357.2, 53.1X, 721.41, 721.42, 721.42, 721.91, 722.1, 722.10, 722.11, 722.2, 722.70, 722.72, 722.73, 724.0X, 724.3, 724.4, 721.1, 722.0, 722.71, 723.0, 723.4, 357.3, 357.8, 357.9, 337.2X, 353.2, 353.3, 353.4, 354.4, 355.7X, 355.9, 729.2, 353.6, 350 |  |  |
|  | Symptoms, signs, and ill-defined conditions | |  |  |  |  |
|  |  | Fatigue |  | 780.71, 780.79 |  |  |
|  |  | Headache |  | 784.0 |  |  |
|  |  | Chest pain |  | 786.5X |  |  |
|  |  | Abdominal pain |  | 789.0X |  |  |
|  |  | Anxiety-related symptoms |  | 780.4, 785.0, 785.1, 786.01, 786.05, 786.09 |  |  |

**Additional File 1.** Definitions of comorbidities of interest
